# Inhibition of spumavirus gene expression by PHF11

**DOI:** 10.1371/journal.ppat.1008644

**Published:** 2020-07-17

**Authors:** Melissa Kane, Vincent Mele, Rachel A. Liberatore, Paul D. Bieniasz

**Affiliations:** 1 Department of Pediatrics, Infectious Diseases Division, UPMC Children’s Hospital of Pittsburgh, Pittsburgh, Pennsylvania, United States of America; 2 Center for Microbial Pathogenesis, University of Pittsburgh School of Medicine, Pittsburgh, Pennsylvania, United States of America; 3 Laboratory of Retrovirology, The Rockefeller University, New York, New York, United States of America; 4 Howard Hughes Medical Institute, The Rockefeller University, New York, New York, United States of America; Loyola University Chicago, UNITED STATES

## Abstract

The foamy viruses (FV) or spumaviruses are an ancient subfamily of retroviruses that infect a variety of vertebrates. FVs are endemic, but apparently apathogenic, in modern non-human primates. Like other retroviruses, FV replication is inhibited by type-I interferon (IFN). In a previously described screen of IFN-stimulated genes (ISGs), we identified the macaque PHD finger domain protein-11 (PHF11) as an inhibitor of prototype foamy virus (PFV) replication. Here, we show that human and macaque PHF11 inhibit the replication of multiple spumaviruses, but are inactive against several orthoretroviruses. Analysis of other mammalian PHF11 proteins revealed that antiviral activity is host species dependent. Using multiple reporter viruses and cell lines, we determined that PHF11 specifically inhibits a step in the replication cycle that is unique to FVs, namely basal transcription from the FV internal promoter (IP). In so doing, PHF11 prevents expression of the viral transactivator Tas and subsequent activation of the viral LTR promoter. These studies reveal a previously unreported inhibitory mechanism in mammalian cells, that targets a family of ancient viruses and may promote viral latency.

## Author summary

Foamy viruses have a unique replication strategy and long evolutionary relationship with their vertebrate hosts that has resulted in wide-spread infection of modern species without any apparent pathogenic consequence. How foamy virus infections are controlled by their hosts is unknown. Here, we demonstrate that infection of a variety of foamy viruses is inhibited by the interferon-stimulated gene product, PHF11, in a species-dependent manner. We show that PHF11 prevents replication by a previously undescribed mechanism, namely by inhibiting gene expression from an internal viral promoter, a conserved and distinct feature of the foamy viruses. Inhibition of early viral gene expression by PHF11 may promote viral latency and the apathogenicity of foamy viruses.

## Introduction

Foamy viruses (FVs) or *spumaviruses* are a subfamily of retroviruses, named for their robust cytopathic effect in tissue culture that is characterized by the formation of large syncytia with small cytoplasmic vacuoles [1]. FV infection is endemic in non-human primates and the presence of infectious isolates or endogenous proviruses in many other species suggests that they may be nearly ubiquitous among animals [2, 3]. Despite their broad tropism and cytotopathicity *in vitro*, FVs appear to be apathogenic *in vivo*, suggesting that they are well controlled by their hosts. Recent evidence has suggested that FVs are the oldest of the retroviruses, and extended periods of coevolution might have enabled FVs to become particularly well adapted to their hosts [3]. The apparent absence of disease during FV infection may be the result of selective pressures on both virus and host to reduce pathogenesis.

The FV replication strategy differs from other retroviruses in several ways [4]. First, reverse transcription of FVs appears to occur late in the FV replication cycle, with infectious particles containing DNA, rather than RNA genomes [5]. Second, FV budding requires direct interaction between the viral Env and Gag proteins, making the production of pseudotyped virions impossible [6]. Third, the FV Pol protein is expressed from a spliced *pol*-specific mRNA—no Gag-Pol fusion protein that is typical of retroviruses is produced [7, 8]. Finally, FVs regulate their gene expression through the use of two promoters, one within the U3 region of the long terminal repeat (LTR), and an additional internal promoter (IP) that is unique to FVs, within the *env* gene. While both promoters are activated by a viral transactivator protein, Tas (or Bel-1) [9], a key feature of this dual reporter configuration is that the internal promoter has modest basal activity in the absence of Tas, and therefore drives initial Tas expression. Conversely, the U3 promoter is strictly Tas-dependent and is virtually silent in its absence. Thus, the basal activity of the IP is crucial for initiating viral gene expression that is subsequently sustained by a Tas-activated positive ‘feedback loop’ that involves both IP and U3 promoters. Notably however, latent infection with transcriptionally silent proviruses is frequent, particularly in blood mononuclear cells, during FV infection [10–12].

Like other retroviruses, FV replication is sensitive to type-I IFN [13–15]. We recently conducted a screen to identify human and rhesus macaque IFN-stimulated genes (ISGs) with antiretroviral activity [16]. Because of the distinct features of FV replication, we further investigated ISGs that uniquely inhibited prototype foamy virus (PFV) infection. Understanding how FV replication is inhibited by IFNs may suggest reasons why FV infection is asymptomatic and how the interplay between FVs and their hosts has resulted in their remarkable evolutionary success.

A screen for ISGs that revealed inhibitors of the ‘early’ (before viral gene expression) steps in PFV replication [16] identified macaque plant homeodomain finger protein 11 (PHF11) as a specific inhibitor (PHF11 was not included in the human ISG library). PHD fingers are specialized zinc fingers commonly found in nuclear proteins that regulate chromatin [17, 18]. PHF11 contains a noncanonical extended PHD (ePHD) domain, which has been suggested to bind dsDNA, but not histones [19, 20]. PHF11 is predominantly expressed in immune cells [21] and has been ascribed multiple functions, including the activation of cytokine genes [15, 21], promotion of specific class switch recombination events [22], and direct participation in DNA repair [23]. Polymorphisms in PHF11 have been linked to elevated total IgE levels, allergic asthma, and eczema [24, 25], although the relevance of these linkages remains controversial [25] and no mechanism has been proposed to explain their role in disease. Here, we demonstrate that PHF11 is a foamy virus-specific inhibitor that attenuates viral gene expression, and may drive latency, by specifically inhibiting the basal activity of the viral IP.

## Results

### PHF11 inhibits foamy virus, but not orthoretrovirus, replication

**Fig 1:**
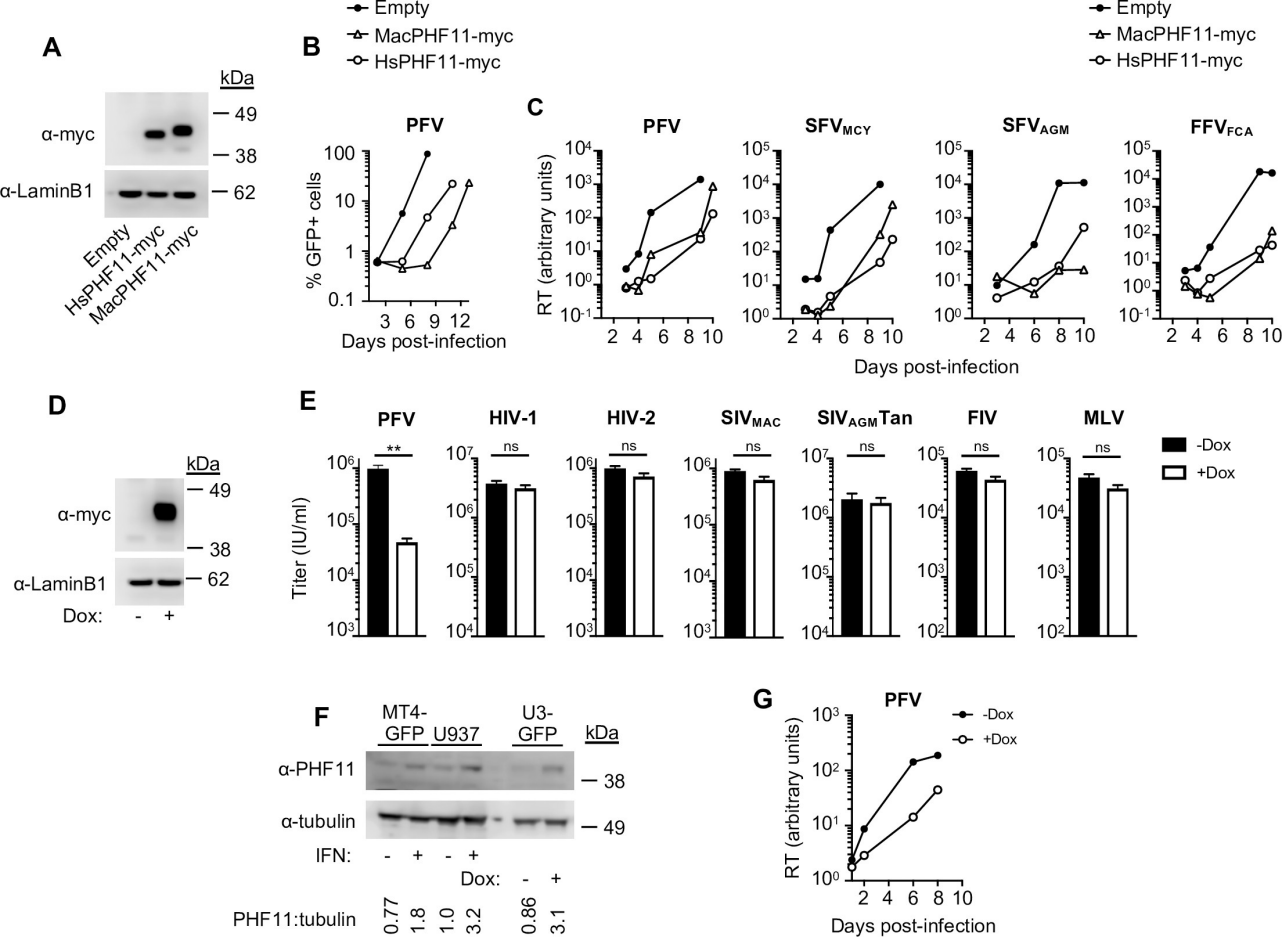
Human and macaque PHF11 specifically inhibit foamy virus replication. (A) Western blot analysis of PHF11-myc and Lamin B1 loading control in U3-GFP cells stably transduced with myc-tagged PHF11 or empty (LHCX) expression vector. Each well was loaded with 10μL of lysate containing 10^4^ cells. (B) Macaque and human PHF11 inhibit PFV replication. Spreading replication (starting MOI = 0.001) in U3-GFP cells stably transduced with myc-tagged PHF11 or empty (LHCX) expression vector. Representative of at least four independent experiments. Infection was measured by FACS analysis of GFP expression. (C) Macaque and human PHF11 inhibit multiple FVs. Spreading replication, measured by a SYBR-PERT reverse transcriptase assay, of PFV, SFV_MCY_, SFV_AGM_, and FFV_FCA_ in PHF11-myc or empty vector expressing U3-GFP cells (starting MOI = 0.01). Representative of at least three independent experiments. Infection was measured via SYBR-Green RT assay. (D) Western blot analysis of doxycycline inducible myc-HsPHF11 and LaminB1 loading control in U3-GFP cells. (E) PHF11 does not inhibit orthoretrovirus infection. Single-cycle infection of U3-GFP cells expressing doxycycline-inducible myc-HsPHF11 with PFV and various GFP-reporter viruses in the presence (white bars) or absence (black bars) of doxycycline (Dox) pretreatment. Titers were calculated based on the percentage of GFP positive cells and are represented as mean + sem of infectious units per ml, n≥3 technical replicates. Representative of at least three independent experiments. ns = not significant; ** = p<0.01. (F) Western blot analysis of PHF11 expression in IFN-treated MT4-LTR-GFP (MT4-GFP), U937 cells, or doxycycline inducible U3-GFP cells and tubulin loading control. The ratio of PHF11 to tubulin levels is indicated below the blots. (G) Spreading replication of PFV (starting MOI = 0.01) in U3-GFP cells expressing doxycycline-inducible untagged-HsPHF11 in the presence (open circles) or absence (filled circles) of doxycycline (Dox) pretreatment. Right: Representative of at least two independent experiments. RT, reverse transcriptase. MOI, multiplicity of infection.

Our prior ISG screens identified PHF-11 as a candidate antiviral ISG [16]. To conveniently quantify wild-type (WT) PFV infection and assess PHF11 antiviral activity, we generated a PFV indicator cell line, which is a clone of HT1080 cells containing an integrated PFV long terminal repeat reporter construct (U3-GFP cells, S1A Fig). In these cells, GFP expression occurs upon PFV infection and is dependent on the PFV transactivator Tas (S1B Fig). We expressed macaque (Mac) or human (Hs) PHF11 proteins in U3-GFP cells (Fig 1A) and tested their effect on PFV spreading replication. Both the macaque and human PHF11 proteins robustly inhibited PFV replication (Fig 1B), confirming PHF11 as a bona fide “hit” from our ISG screen.

PFV (SFV_CPZ_) is produced from an infectious clone isolated from an infected human cell culture but is likely of chimpanzee origin [26, 27]. To determine whether sensitivity to PHF11 is an incidental feature of this clone, we assessed the PHF11 sensitivity of FV isolates from two Old World monkeys, namely Formosan rock macaques [simian foamy virus-1 (SFV-1, SFV_MAC_, or SFV_MCY_)] and African green monkeys (SFV-3, SFV_AGM_, or SFV_CAE_), and one from domestic cats (FFV or FFV_FCA_). Since Tas-dependent activation of foamy virus gene expression is species-specific [26, 28] and only PFV infection induces GFP expression in U3-GFP cells, we used a virion-associated reverse transcriptase (RT) activity assay to measure replication of the various FV species. Notably, both human and macaque PHF11 inhibited the replication of all four foamy viruses (Fig 1C).

Next, we generated U3-GFP cells expressing doxycycline-inducible PHF11 (Fig 1D) and monitored their growth and viability over time in the presence or absence of doxycycline. This analysis revealed that induction of PHF11 expression did not affect cellular growth rate or viability (S2A and S2B Fig). We then challenged these cells with PFV and a variety of GFP-encoding retroviruses, including human immunodeficiency viruses (HIV-1 and HIV-2), simian immunodeficiency viruses (SIV_MAC_ and SIV_AGM_Tan), feline immunodeficiency virus (FIV), and murine leukemia virus (MLV) and measured GFP expression after 48 hours (Fig 1E). Although WT PFV is replication competent, the 48-hour time-point used in these experiments represents a single cycle of infection, since the addition of raltegravir 24 hours after infection did not alter GFP expression (S3 Fig). While PHF11 inhibited PFV infection ~20-fold, none of the other retroviruses were affected (Fig 1E), indicating that PHF11 is a specific inhibitor of FVs.

PHF11 is predominantly expressed in immune cells [15, 21] and was not detectable in IFNα-treated HT1080-derived U3-GFP cells (S4 Fig). Previous microarray analyses indicated that IFNα-treatment upregulates PHF11 expression in MT4-LTR-GFP (MT4 cells containing an HIV-1 LTR reporter) [16] and U937 [29] cells but FV did not replicate efficiently in these cell lines. However, expression of PHF11 in U3-GFP cells at levels comparable to those in IFNα-treated MT4-LTR-GFP and U937 cells (Fig 1F) inhibited PFV replication therein (Fig 1G), indicating that levels of PHF11 found endogenously in MT4 and U937 cells are capable of inhibiting PFV infection.

### Mammalian PHF11 proteins are variable and differ in their FV-inhibitory activity

**Fig 2:**
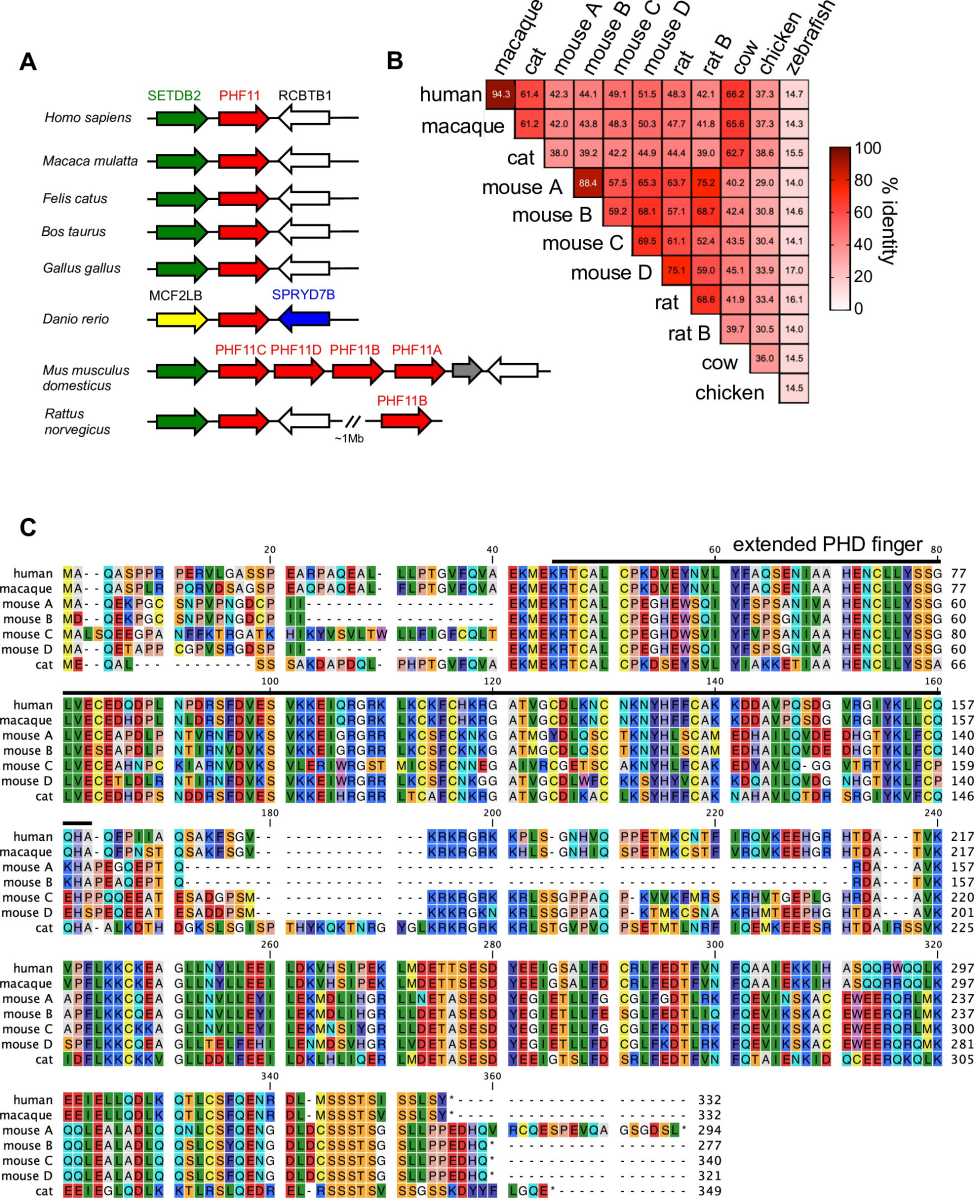
The PHF11 locus and alignment of PHF11 genes: significant species dependent variability in PHF11 genes. (A) Diagram of the PHF11 locus in the indicated species. (B) Heat map of percent identity of PHF11 proteins in vertebrates. (C) Amino acid alignments of human, macaque, mouse, and cat PHF11 proteins used in this investigation, with the extended PHD finger indicated by a bold line.

**Fig 3:**
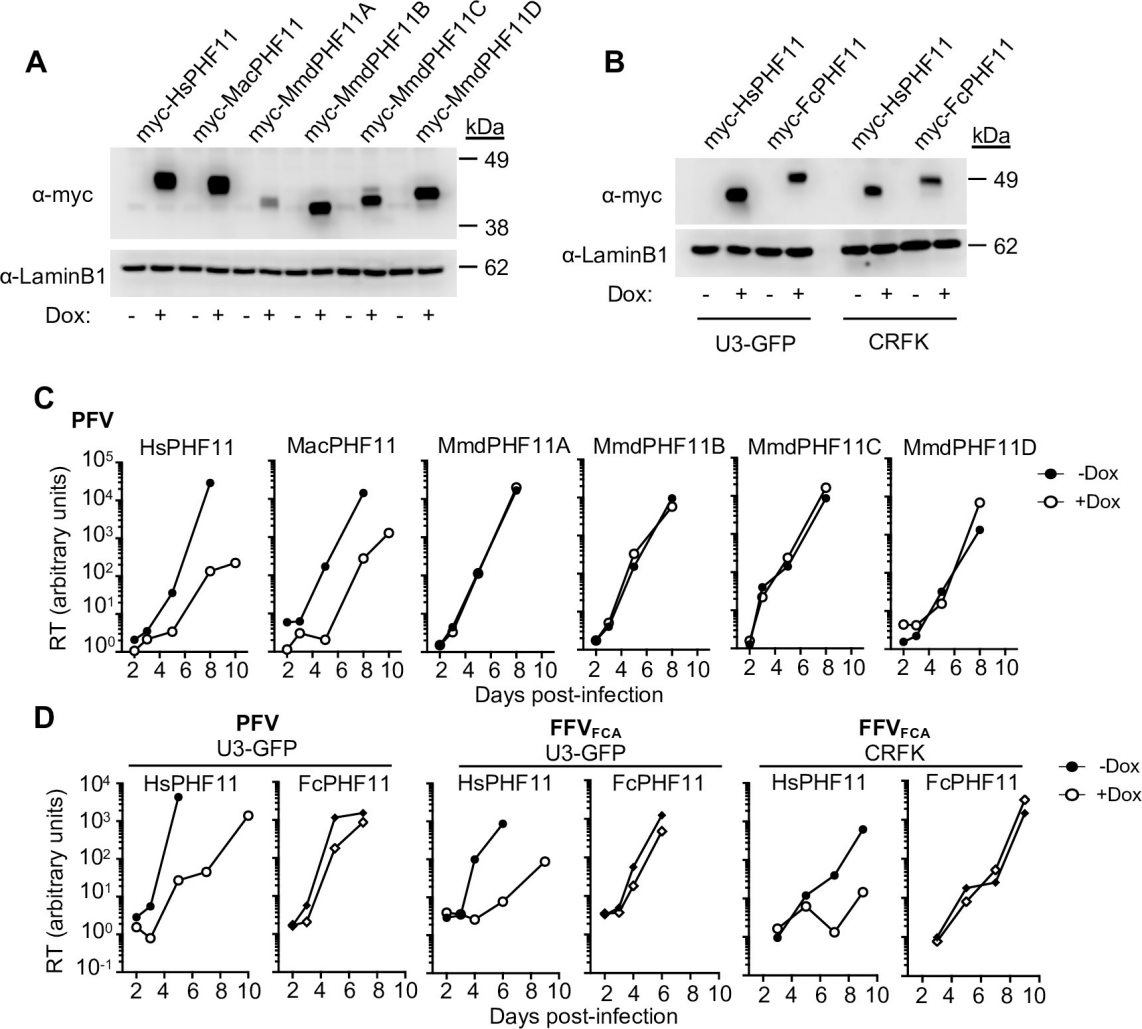
Antiviral activity of mammalian PHF11 proteins: Murine and feline PHF11 genes do not inhibit FV replication. (A) Western blot analysis of doxycycline inducible human, macaque and mouse myc-PHF11 and LaminB1 loading control in U3-GFP cells. (B) Western blot analysis of doxycycline inducible human and cat myc-PHF11 and LaminB1 loading control in human U3-GFP and feline CRFK cells. (C) Mouse PHF11 genes do not inhibit PFV replication. Spreading replication of PFV (starting MOI = 0.01) in U3-GFP cells expressing doxycycline-inducible myc-tagged human (Hs), macaque (Mac) or mouse (Mmd) PHF11 in the presence (white bars) or absence (black bars) of doxycycline (Dox)pretreatment. Infection was measured via SYBR-Green RT assay. (D) Feline PHF11 does not inhibit PFV or FFV replication. Spreading replication of PFV or FFV_FCA_ (starting MOI = 0.05) in U3-GFP or CRFK cells expressing doxycycline-inducible myc-tagged Hs (circles) or feline (Fc) (diamonds) PHF11 in the presence (open symbols) or absence (filled symbols) of Dox. Representative of at least three independent experiments. Infection was measured via SYBR-Green RT assay. RT, reverse transcriptase. MOI, multiplicity of infection.

We next investigated whether PHF11 proteins from other species also exhibit antiviral activity. The *Phf11* gene appears to be unique to vertebrates [23] so we compared the sequences of *Phf11* genes from several mammalian species, as well as chickens (*Gallus gallus*) and zebrafish *(Danio rerio*) (Fig 2). Interestingly, we found that while humans and macaques have a single *Phf11* gene there has been an expansion of the *Phf11* locus in rodents, and in particular in mice *(Mus musculus domesticus*), which have four *Phf11* genes (Fig 2A). We also found that PHF11 proteins are notably variable in sequence, even among mammals: while proteins encoded by the human and macaque *Phf11* genes share 94% identical amino acids, they are only 61% identical to cat (*Felis catus*) PHF11 and only 42–50% identical to the four mouse PHF11 proteins (Fig 2B and 2C). To investigate whether this sequence divergence affected antiviral activity, we expressed the mouse (Mmd) and feline (Fc) PHF11 proteins in U3-GFP or CRFK cells (Fig 3A and 3B) and measured their effect on PFV in a single-cycle and spreading infection. None of the mouse PHF11 genes inhibited PFV replication (Fig 3C and S5A Fig), and the feline PHF11 exhibited little activity against PFV or FFV_FCA_ infection in U3-GFP cells (Fig 3D and S5B Fig). Expression of antiviral genes in cells from other species has the potential to alter their activity due to differences in required cofactors. However, while human PHF11 retained its inhibitory activity against FFV_FCA_ in CRFK cells, feline PHF11 had no effect on FFV_FCA_ replication (Fig 3D). Thus, although the primate PHF11 proteins tested here inhibited the replication of both primate and feline foamy viruses, feline PHF11 exhibited little or no activity against either virus.

To identify the region(s) of PHF11 that determine antiviral activity, we generated chimeric human-feline PHF11 proteins (S5C Fig) that were all well expressed (S5D Fig) and challenged U3-GFP cells expressing each of the chimeric PHF11 proteins with PFV in single-cycle infection assays (S5E Fig). Replacing the N-terminal domain of the active HsPHF11 with the corresponding region of the inactive FcPHF11 gave a chimera (Hs(FcNTD)) that retained antiviral activity, while the reciprocal chimera (Fc(HsNTD)) largely lacked antiviral activity (S5E Fig). Replacing either the PHD or C-terminal domains (CTD) of HsPHF11 with FcPHF11 generated chimeras (Hs(FcPHD) and Hs(FcCTD)) that lacked antiviral activity. Conversely, transfer of either the PHD or CTD of HsPHF11 to FcPHF11 gave chimeras (Fc(HsPHD) and Fc(HsCTD)) that both inhibited PFV infection (S5E Fig). Thus, while both the PHD and CTD domains of HsPHF11 contain determinants that were sufficient to confer antiviral activity on the otherwise inactive FcPHF11, both the PHD and CTD domains of FcPHF11 were sufficient to attenuate antiviral activity when replacing the corresponding domains in HsPHF11. Overall these data suggest that all three PHF11 domains (NTD, PHD and CTD) contain determinants that contribute to PHF11 antiviral activity.

### PHF11 inhibits a post-integration step in FV infection

**Fig 4:**
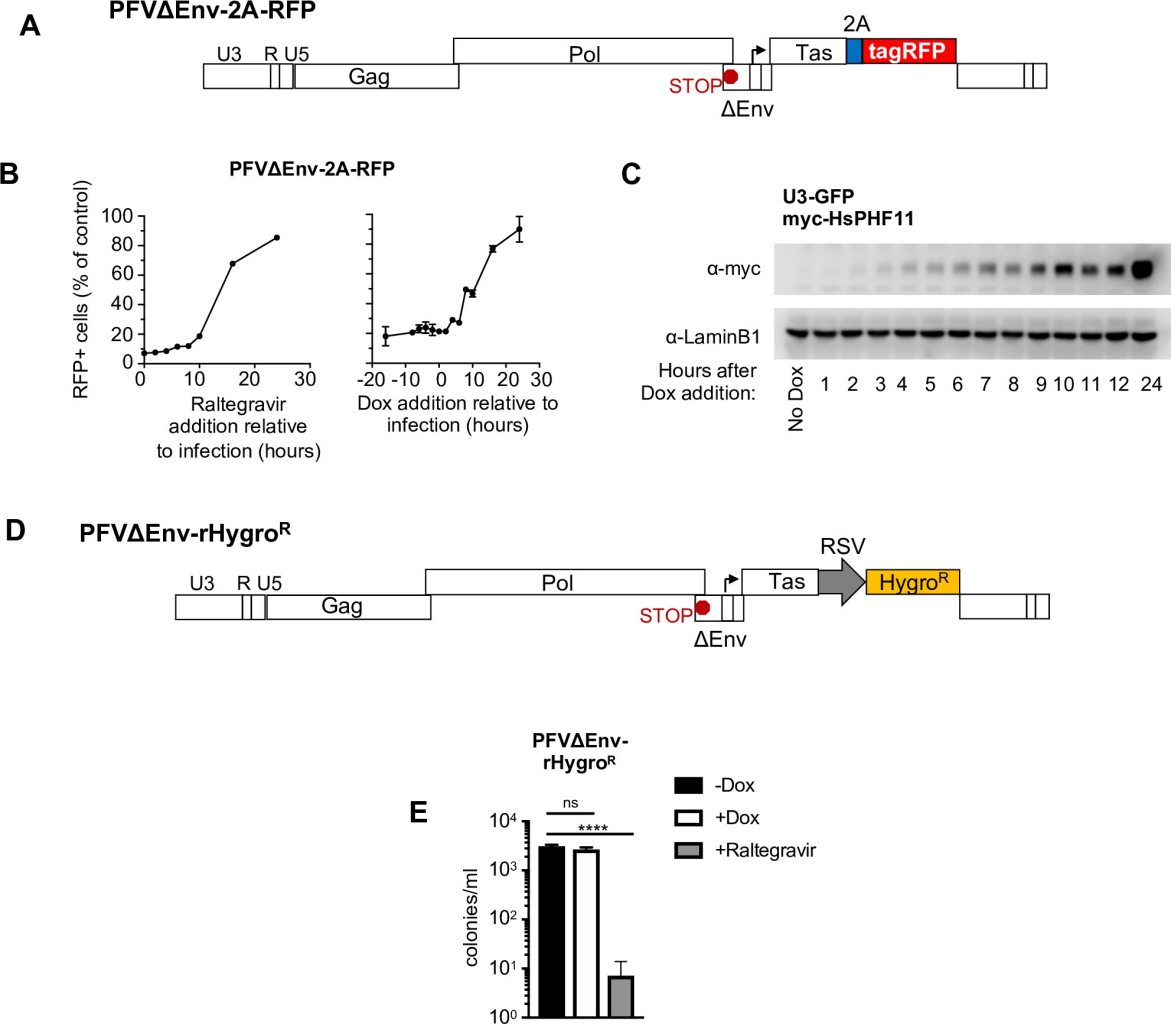
PHF11 inhibits FV infection post-integration. (A) Schematic representation of the PFVΔEnv-2A-RFP reporter vector. The location of the internal promoter is indicated by an arrow. The premature stop codon introduced into the ΔEnv constructs is indicated by a red octagon. (B) PFV is not inhibited by PHF11 once infection is established. Time-course of the sensitivity of PFVΔEnv-2A-RFP reporter virus to raltegravir (left) or doxycycline-inducible myc-PHF11 (right). Infectivity is plotted as the number of RFP positive cells as a percentage of the number of cells infected in the absence of raltegravir or doxycycline at the same time. Left: PFV sensitivity to raltegravir is lost over time, indicating that integration is completed by the majority of viruses. Right: PFV also loses sensitivity to PHF11 over time. (C) Western blot analysis of indicated doxycycline (Dox) inducible myc-HsPHF11 and LaminB1 loading control in U3-GFP cells, with Dox added at the indicated time-points. (D) Schematic representation of the PFVΔEnv-rHygroR reporter vector that contains an RSV promoter driven hygromycin resistance cassette. The premature stop codon introduced into the ΔEnv constructs is indicated by a red octagon. (E) PHF11 does not inhibit integration-dependent hygromycin-resistant colony formation. Infection of U3-GFP cells expressing doxycycline-inducible myc-HsPHF11 in the presence (white bars) or absence (black bars) of doxycycline (Dox) pretreatment with the indicated hygromycin-B reporter virus. Infection was measured by counting the number of hygromycin-B-resistant colonies following 10–14 days of selection in hygromycin post infection. Titers are mean + SEM, n = 4 technical replicates. Representative of four independent experiments. ns = not significant; **** = p≤0.0001.

To elucidate which step in the FV replication cycle is inhibited by PHF11, we first determined the timing with which sensitivity to inhibition by PHF11 was lost during a single cycle of infection. To ensure that only a single-cycle of infection occurred in these experiments, we constructed an envelope-deficient Tas-2A-tagRFP reporter virus (PFVΔEnv-2A-RFP) in which both Tas and tagRFP were expressed from the FV internal promoter (Fig 4A) and generated virions by co-transfection of the envelope-deficient proviral plasmid with a PFV-Env expression plasmid. As expected, PFVΔEnv-2A-RFP sensitivity to an integrase inhibitor, raltegravir, diminished with time after infection and this analysis indicated that the majority of incoming virions completed integration (inferred by loss of raltegravir sensitivity) at 10–20 hours post-infection (Fig 3B). Next, we added doxycycline to U3-GFP cells stably transduced with a doxycycline inducible myc-tagged PHF11 at various time points relative to PFVΔEnv-2A-RFP infection. Western blot analysis of myc-PHF11 expression revealed that PHF11 became detectable three to four hours after doxycycline addition, and levels increased over the ensuing ~7-24h (Fig 4C). Doxycycline addition (PHF11 induction) was nearly fully active in inducing FV inhibition when applied 8h after infection, but its activity began to diminish thereafter (Fig 3B). Thus, given the 3 or more hours of delay in the onset of PHF11 expression relative to doxycycline addition, this result suggested that PHF11 acted to suppress FV replication at or around the time of integration, but was ineffective thereafter.

Therefore, to directly measure the effect of PHF11 on PFV integration, we generated an envelope-deficient PFV construct containing a hygromycin B-resistance gene (Hygro^R^) expressed from the RSV promoter (PFVΔEnv-rHygro^R^, Fig 4D). Cells were infected with these virions at a MOI of less than 0.01 to ensure that each hygromycin-resistant colony would represent a single integration event, and we measured the number of colonies that formed following infection in the presence or absence of PHF11. Hygromycin-resistant colony formation in this infection assay was dependent on integration, since colony formation was nearly completely abolished in the presence of raltegravir (Fig 4E). Notably, PHF11 expression did not diminish the number of hygromycin-resistant colonies (Fig 4E), indicating that integration was unaffected by PHF11.

### PHF11 inhibits FV gene expression

Because PHF11 had no detectable effect on PFV integration, but the timing of its action indicated an inhibitory effect at or around the time of integration, we next investigated whether PHF11 inhibited FV gene expression. FV transcription occurs from both the LTR promoter, and from an internal promoter (IP) upstream of the regulatory and accessory genes [28]. Tas activates FV transcription by directly binding to DNA target sequences in both the LTR and IP, despite little sequence relatedness between the two promoters [30]. Because the IP has higher basal activity in the absence of Tas, the IP initiates a cascade of FV gene expression [31]. As the level of Tas protein translated from IP-initiated transcripts increases, the IP is further activated, the LTR promoter becomes activated, and the viral structural genes are expressed [28].

**Fig 5:**
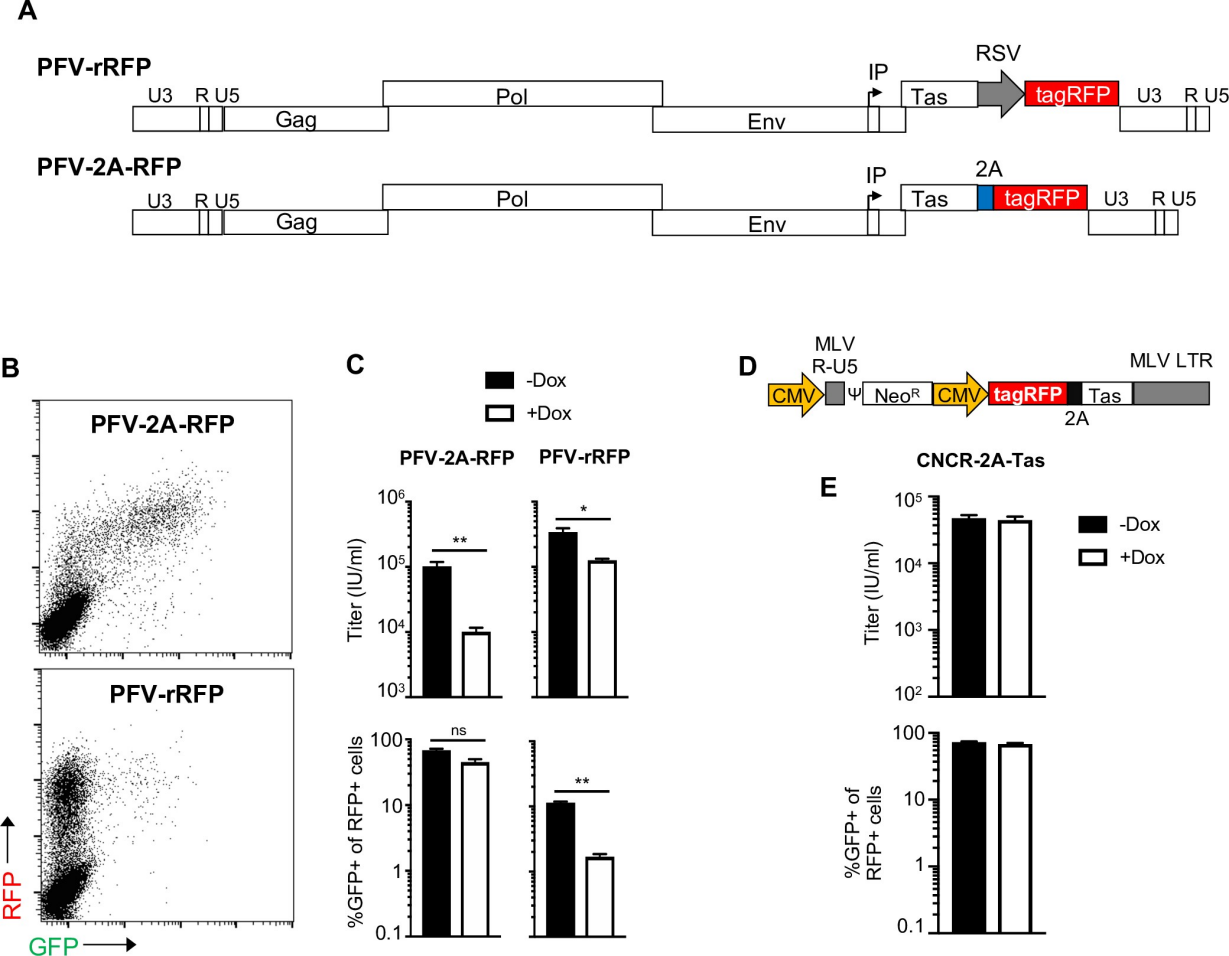
PHF11 inhibits FV gene expression, but not Tas transactivation. (A) Schematic representation of the PFV-rRFP and PFV-2A-RFP reporter viruses. The location of the internal promoter (IP) is indicated by an arrow. Tag RFP is expressed using either an RSV promoter or the IP, linked to Tas (B) Sample FACS plots illustrating GFP and RFP expression in U3-GFP cells infected with PFV-rRFP and PFV-2A-RFP reporter viruses. (C) PHF11 inhibits PFV gene expression. Infection of U3-GFP cells expressing doxycycline-inducible myc-HsPHF11 with the indicated PFV-rRFP and PFV-2A-RFP reporter virus in the presence (white bars) or absence (black bars) of doxycycline (Dox) pretreatment. The titers, as measured based on the number of RFP positive cells is indicated in the upper panels, and the proportion of RFP-positive cells that were also GFP positive is indicated in the lower panels. (D) Schematic representation the MLV CNCR-based reporter virus expressing RFP-2A-Tas. (E) PHF11 does not inhibit transactivation of the U3 promoter when Tas expressed from the CMV promoter. Infection of U3-GFP cells expressing doxycycline-inducible myc-HsPHF11 in the presence (white bars) or absence (black bars) of pretreatment with doxycycline (Dox) with the MLV CNCR-based reporter virus containing RFP-2A-Tas. Top: viral titer as determined by percentage of RFP positive cells. Bottom: percentage of RFP positive cells that are also GFP positive (from samples with 10–40% RFP positive cells). Titers are mean + SEM n = 3 technical replicates. Representative of four independent experiments.

To test whether PHF11 inhibits FV gene expression, we used U3-GFP reporter cells and two FV reporter constructs in which tagRFP expression was dependent on either the FV IP (PFV-2A-RFP) or on an introduced RSV promoter (PFV-rRFP) (Fig 5A). Notably, in PFV-2A-RFP infected cultures, the vast majority of RFP-positive U3-GFP cells also expressed GFP (Fig 5B and 5C). This is as expected, because the PFV-encoded RFP reporter is directly linked to Tas and the U3-GFP reporter is dependent on Tas expression. However, in cells infected with PFV-rRFP, only ~10% of RFP positive cells also expressed GFP (Fig 5B and 5C). This discrepancy suggests that a large fraction of the PFV-rRFP infected cells do not express Tas, and that expression of tagRFP from the RSV promoter (in RFP-positive, GFP-negative cells) indicates PFV-rRFP infected cells that harbor either latent and/or defective proviruses that do not express Tas. We then compared the sensitivity of PFV-2A-RFP and PFV-rRFP reporter viruses to PHF11, as measured by the percentage of RFP-positive cells. Titers of PFV-2A-RFP were reduced 10-fold by PHF11, while titers of PFV-rRFP were reduced only 2.7-fold (Fig 5C). However, when we examined expression of the U3-GFP reporter in RFP-positive (infected) cells, most of the PFV-2A-RFP infected, RFP-positive cells were also GFP positive (Fig 5C, lower left), even when PHF11 was expressed. Conversely, in PFV-rRFP infected cells, PHF11 reduced the proportion of RFP positive cells that were also GFP-positive from ~10% to ~1% (Fig 5C, lower right). Put another way, PHF11 inhibited the activation of the U3-GFP reporter in cells that were demonstrably infected by PFV-rRFP. This result strongly suggested that PHF11 inhibits gene expression driven from either or both the IP or U3 promoter(s). The modest reduction in RFP expression in PFV-rRFP infected cells when PHF11 was expressed (Fig 5C, upper right) might be due to marginal sensitivity of the RSV promoter to PHF11, or by some fraction of RFP+ cells arising from transcripts initiated at the IP rather than the RSV promoter.

To directly test whether PHF11 inhibits Tas-dependent activation of the PFV U3 promoter we delivered Tas to U3-GFP cells in the context of different retroviral vector, based on murine leukemia virus (MLV) [32, 33], engineered to express RFP-2A-Tas from a CMV promoter (CNCR-2A-Tas, Fig 5D). In this case, infectious CNCR-2A-Tas titers, as determined by RFP expression, were unaffected by PHF11 expression (Fig 5E) as expected, since MLV is insensitive to PHF11 (Fig 1E). Activation of the PFV U3 promoter by the Tas protein expressed by CNCR-2A-Tas was efficient, as the vast majority of RFP positive cells also expressed GFP (Fig 5E). Notably, PHF11 had no effect on Tas-activated, U3-driven GFP expression in CNCR-2A-Tas infected U3-GFP cells (Fig 5E). The result with CNCR-2A-Tas contrasts clearly with that obtained with PFV-rRFP and PFV-2A-Tas (Fig 5C) and suggests that PHF11 has no effect on U3 driven transcription when Tas is expressed in the context of another retrovirus. These results should be interpreted with caution however, as the CMV promoter may drive levels of Tas expression that are greater than those present during FV infection. Nevertheless, this finding suggested that the effects of PHF11 on replication, and on Tas-dependent GFP expression PFV-rRFP infected U3-GFP cells, is an indirect result of reduced Tas expression from the IP.

**Fig 6:**
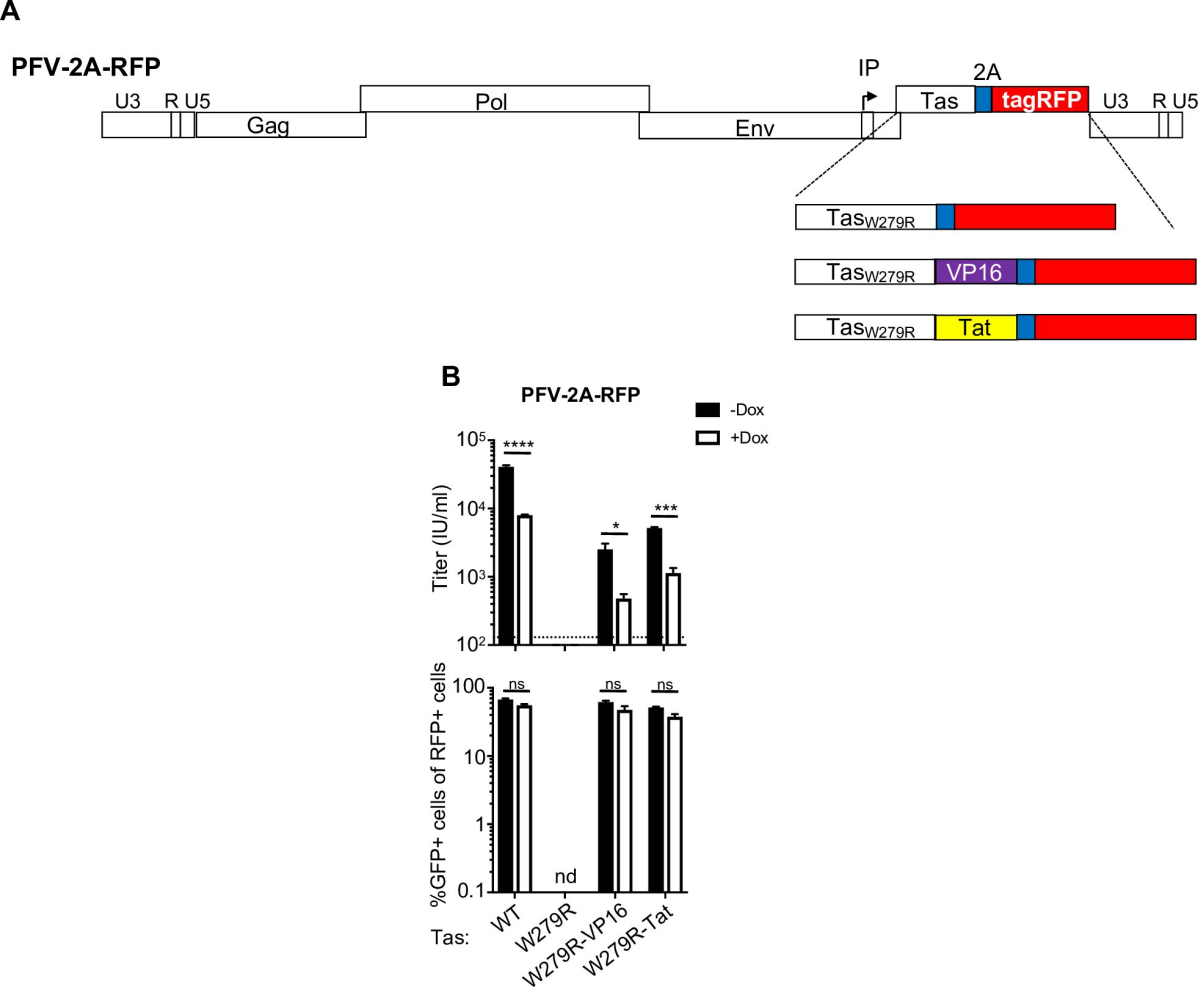
PHF11 does not specifically target the Tas activation domain. (A) Schematic of Tas mutant and Tas-transactivator fusion-expressing reporter viruses. TasW297R mutant and fusions were inserted into PFV-2A-RFP. VP16 = HSV-1 VP16 activation domain; Tat = HIV-1 NL4.3 Tat activation domain (Tat N-terminal 48 amino acids). (B) Infection of U3-GFP cells with Tas mutant or Tas-transactivator fusion-expressing PFV-2A-RFP reporter viruses. The top panel indicates viral titer as determined by percentage of RFP positive cells, the bottom panel indicates the percentage of RFP positive cells that were also GFP positive (from samples with 10–60% RFP positive cells). Titers are mean + SEM, n = 3 technical replicates. Representative of at least three independent experiments. nd = not detectable; ns-not significant; * = p<0.05; ** = p<0.01; *** = p<0.001; **** = p≤0.0001.

PFV Tas directly binds to the proviral LTR or the IP via its N-terminal DNA-binding domain (amino acids ~80–210), and activates transcription through its C-terminal activation domain (amino acids ~250–290) [34]. The DNA-binding and activation domains can be functionally separated, and fusion of the activation domain to a heterologous DNA binding domain such as yeast GAL4, can activate transcription in mammalian, avian, and yeast cells [35]. The Tas activation domain belongs to the same functional class of activation domains that includes p53 and the herpes simplex virus VP16 protein (acidic class type IIB) [36, 37]. To test whether the inhibition exerted by PHF11 is specific to the Tas activation domain, we introduced a point mutation in Tas (W279R, Fig 6A) that eliminates transcriptional activation activity [[36] and Fig 6B] and tested whether this mutant could be rescued from PHF11 inhibition by fusing Tas to a heterologous transactivator domain (Fig 6A). We selected (i) the transactivator domain of VP16, which promotes both initiation and elongation [37], and (ii) residues 1–48 of HIV-1 Tat (that can function as a type IIA transactivator when fused to a DNA binding domain and promote elongation [37, 38]). Both Tas_W269R_-transactivator fusions partially rescued infectivity of the PFV_W269R_ mutant virus but both viruses retained sensitivity to PHF11 (Fig 6B). Thus, PHF11 does not inhibit FV replication via specific interference with the function of the Tas activation domain.

### PHF11 inhibits the activity of the PFV internal promoter

**Fig 7:**
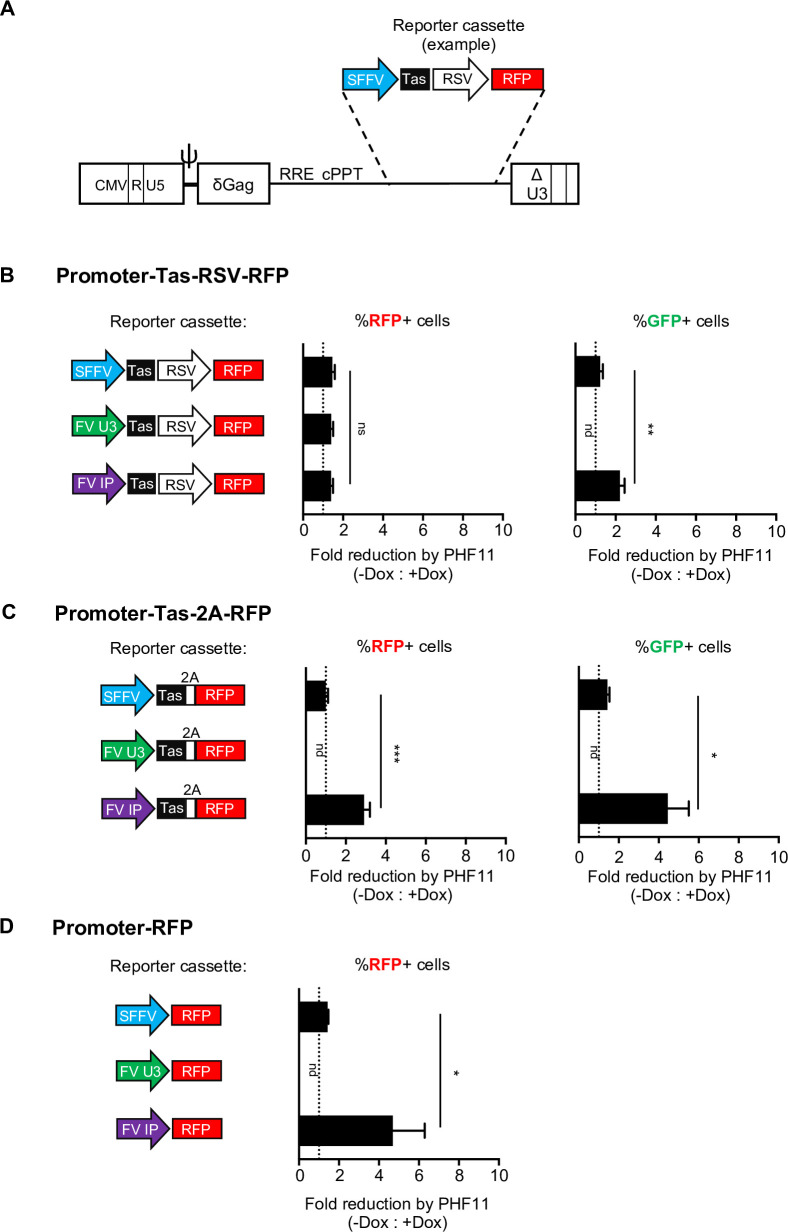
PHF11 inhibits expression from the FV internal promoter. (A) Experimental design: Schematic of the CSGW construct on which the reporter vectors are based, with an example reporter cassette. (B-D) U3-GFP cells expressing doxycycline-inducible myc-HsPHF11 in the presence or absence of doxycycline (Dox) pretreatment were transduced with the indicated reporters. Left panels: Promoters directing Tas expression and RFP expression and diagrams of promoter, viral, and reporter elements. Middle panels: fold reduction in the percentage of RFP positive cells in the presence of doxycycline compared to absence of doxycycline. Right panels: fold reduction in the percentage of GFP positive cells in the presence of doxycycline compared to absence of doxycycline. Fold changes are presented as mean + SEM, n≥5 technical replicates combined from two independent experiments. Representative of four total independent experiments. nd = not detected; ns = not significant; * = p<0.05; ** = p<0.01; *** = p<0.001. (B) RFP expression from the RSV promoter, Tas expression from the SFFV, U3, or IP promoters. RFP expression is Tas-independent, GFP expression is Tas-dependent. (C) Tas expression from the SFFV, U3, or IP promoters, RFP expression is linked to Tas via a 2A cleavage site. SFFV-Tas-2A-RFP: RFP expression is Tas-independent, GFP expression is Tas-dependent. U3-Tas-2A-RFP: both RFP and GFP expression are Tas-dependent. IP-Tas-2A-RFP: RFP expression is both Tas-dependent and -independent, GFP expression is Tas-dependent. (D) RFP expression from the SFFV, U3, or IP promoters without Tas.

We sought to resolve the apparent discrepancy in the effects of PHF11 on the U3-GFP reporter following infection with PFV-rRFP versus CNCR-2A-Tas. To accomplish this, we developed an approach to directly measure the effect of PHF11 on reporter gene expression from individual integrated viral promoter(s). We generated an array of packageable constructs based on an HIV-1 vector, CSGW [39], which contains a self-inactivating LTR, allowing assessment of integrated reporter gene expression from a promoter of choice (Fig 7A). In these constructs, Tas and/or RFP were expressed from (i) the spleen focus forming virus (SFFV) U3 promoter, (ii) RSV U3 promoter, (iii) the PFV U3 promoter or (iv) the PFV internal promoter (IP) (Fig 7A–7D) The inocula were normalized based on RT activity to ensure equivalent levels of transduction, and introduced into U3-GFP cells. Levels of both RFP (expressed by the incoming construct) and GFP (expressed by Tas *trans*-activation of the pre-existing U3-GFP reporter) were measured and converted into a fold reduction (or fold change) comparing absence or presence of doxycycline (Fig 7, raw data is presented in S5 Fig).

**Fig 8:**
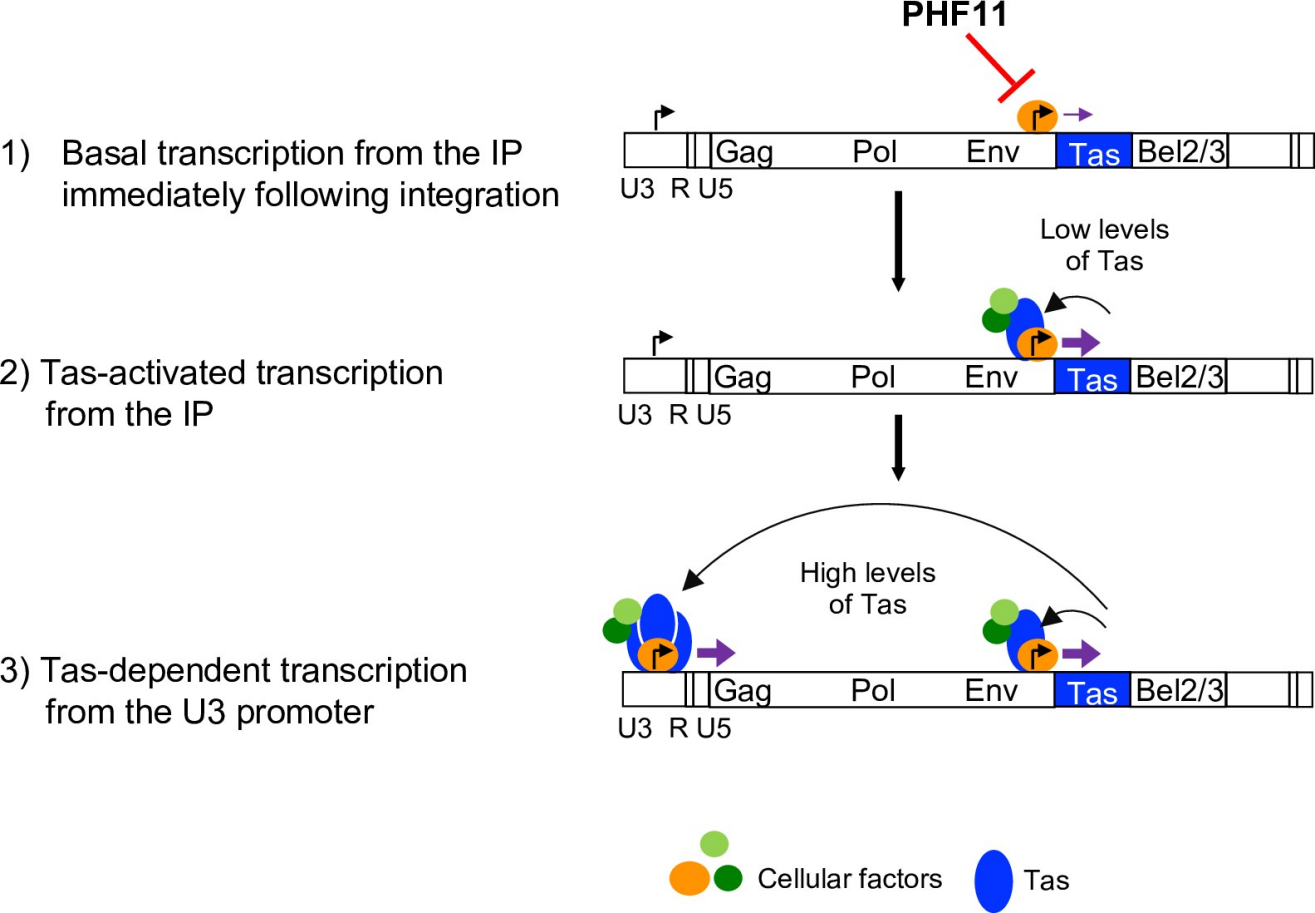
Model of FV gene expression and proposed mechanism of PHF11 inhibition. Cartoon of the proviral genome with relative locations of the viral genes indicated. Locations of the U3 and internal promoter (IP) are indicated by black arrows. Transcription is indicated by purple arrows, with increased size indicating relative levels of transcription 1) Immediately after integration, Tas-independent basal transcription from the IP is initiated by unknown cellular factors [31]. This results in low levels of Tas expression sufficient to 2) stimulate Tas-dependent transcription from the IP and initiate a ‘positive-feedback’ loop that produces high levels of Tas sufficient to 3) stimulate the LTR U3 promoter, expression of Gag, Pol, and Env, and production of full-length viral genomes. Both basal transcription from the IP and Tas-dependent transcription utilize unknown cellular factors [51]. PHF11 inhibits viral gene expression by preventing the initial Tas-independent basal transcription from the IP, preventing Tas expression from reaching a level that is sufficient for subsequent IP and LTR transactivation.

When RFP or Tas were expressed from the SFFV promoter, PHF11 had no effect on either RFP or Tas-activated GFP expression (Fig 7B–7D and S6 Fig), indicating that PHF11 did not affect the SFFV or Tas-activated U3 promoters. RSV promoter-dependent RFP expression was also unaffected by PHF11 (Fig 7B). Conversely, when both Tas and RFP were under the control of the internal promoter (IP-Tas-2A-RFP), expression of both RFP and GFP were inhibited by PHF11 (Fig 7C), indicating that PHF11 inhibits expression from the IP, and thus indirectly, Tas activation of the U3-GFP construct. Consistent with this notion, when Tas, but not RFP expression was under the control of the internal promoter (IP-Tas-RSV-RFP), PHF11 reduced the number of GFP+ cells, but not the number of RFP positive cells (Fig 7B and S6 Fig). Notably, neither RFP nor GFP were detected when Tas and/or RFP were under the control of the U3 promoter alone, irrespective of PHF11 expression (Fig 5B–5D), Thus, basal activity from the U3 promoter is insufficient to generate levels of Tas that are sufficient for ‘feedback’ U3 transactivation. Conversely, low levels of RFP expression were detected from the IP, even in the absence of Tas (Fig 7D). These results are consistent with previous reports indicating that FV transcription is initiated at the internal promoter, which has higher basal activity and higher affinity Tas-binding sites than U3 [30, 31]. Importantly, basal expression from the IP, in the complete absence of Tas, was inhibited by PHF11 (Fig 7D and S6 Fig). This finding suggests that antiviral activity of PHF11 is the result of a Tas-independent effect that prevents expression of Tas, and subsequent initiation of a positive feedback loop that drives expression of Tas from the IP and other viral proteins from the U3 promoter (Fig 8).

## Discussion

Here, we demonstrate that human and macaque PHF11 specifically inhibit the replication of multiple viruses from the spumavirus subfamily, while all orthoretroviruses tested were insensitive. The inhibitory activity of PHF11 is species-dependent, with human and macaque PHF11 displaying activity against both primate and non-primate FVs, while feline PHF11 and all four murine PHF11 genes have little or no activity against the four FV strains tested herein. This type of species-specificity is distinct from that exhibited by retroviral restriction factors that are efficiently antagonized by viral accessory genes (such as APOBEC3G/Vif and Tetherin/Vpu [40]), or evaded (such as TRIM5α-mediated inhibition of SFV in New World monkeys, which inhibit replication of SFVs from other species but not their own [41]) Rather, the species dependent inhibition exhibited by PHF11 is akin to that exhibited by proteins such as Mx2, which appears to have more recently acquired anti-lentiviral activity and is not counteracted by a viral protein [42] Interestingly, there has been an expansion of the *Phf11* locus in rodents, resulting in two to four unique *Phf11* genes in rats and mice respectively, all of which are divergent from human *Phf11* and from each other. While there are no known modern exogenous foamy viruses that naturally infect mice, their genomes contain endogenous elements distantly related to modern spumaviruses [43]. Although speculative, it is possible that ancient spumavirus-related viruses could have been pathogenic and driven the proliferation and diversification of murine *Phf11* genes. Alternatively, the FV-inhibitory activity of PHF11 may be a recently acquired function that is unique to primates. This latter notion is consistent with the apparent lack of activity in both murine and feline PHF11 proteins, despite the prevalence of FFV infections [2], and the additional cellular functions unrelated to viral infection that have been ascribed to human and mouse PHF11 genes [15, 21–23]. Importantly, the lack of antiviral activity of feline PHF11, and apparent lack of disease in FFV infected cats indicates that FV replication can be controlled in the absence of PHF11. Other feline genes may possess similar antiviral activity, or FFV replication may be controlled via entirely divergent mechanisms.

By studying the FV transcriptional elements in isolation, and in the presence or absence of Tas we could demonstrate that PHF11 inhibits basal expression from the IP, thereby preventing Tas expression from reaching a level that is sufficient for subsequent IP and LTR transactivation (Fig 8). This conclusion is consistent with our finding that the timing of PHF11 antiviral action is around the time of integration, yet PHF11 does not inhibit integration itself. Once infection is established, and Tas has been expressed, PHF11 no longer exhibits antiviral activity. Thus, by interrupting the initiation of a Tas-driven positive feedback loop, PHF11 may drive FVs into a latent rather than transcriptionally active state. This inhibitory mechanism is distinct from previously reported inhibitors that specifically inhibit Tas-dependent transcriptional activation, including nuclear factor I (NFI) [44] that inhibits Tas-dependent transcription by binding to the FV IP promoter as well as N-Myc interactor (Nmi) [45], p53-induced RING-H2 (PIRH2) [46], and promyelocytic leukaemia (PML) [47] that act by binding to and sequestering Tas.

Notably, PHF11 inhibits highly divergent FVs (PFV and FFV_FCA_). Comparison of the FV Tas proteins reveals only low levels of sequence identity, and while a small portion of the activation domain of Tas is conserved among the simian FVs, this sequence is absent in FFV_FCA_ [2]. Furthermore, Tas response elements (TREs) in both the LTR and the IP exhibit no obvious sequence conservation (except for some similarity between the IP-TREs of PFV and SFV_MCY_) [2]. Cell-type specific differences in both basal and Tas-dependent transcription from the two promoters are evident, suggesting that distinct cellular factors may be involved in each stage of FV gene expression [48, 49]. How then can the breadth of anti-FV activity of human and macaque PHF11 be explained? Notably the IP is a feature present in all FVs, including the ancient endogenous sloth (SloEFV) and coelacanth FV (CoeEFV), the latter of which is estimated to be some 400 million years old [2, 50]. A highly conserved sequence element encompassing and extending beyond the TATA box is present in FV IPs, implying an importance for these sequences in the initiation of FV transcription, while sequences surrounding the U3 TATA box are less well conserved [2]. It is therefore likely that the spumaviruses utilize a common mechanism, and perhaps common cellular factor(s) for the initiation of basal transcription from the IP. Thus, PHF11 may target these conserved features the IP directly or indirectly to inhibit FV transcription.

The evolutionary success of the spumaviruses may result from a replication strategy that does not adversely affect host health, but also enables efficient viral transmission. This strategy appears to involve extremely low levels of replication in infected animals [51], with primarily latent infection of circulating blood cells [12]. While latency in FV infection has not been extensively studied, robust FV transcription is observed in cultured fibroblast cell lines while much lower levels of viral gene expression are found in infected cells of hematopoietic origin [48, 51]. The findings reported here evoke the possibility that PHF11 plays a role in limiting levels of FV replication *in vivo* by preventing the initiation of viral gene expression, in so doing an apparently ‘antiviral’ protein may facilitate persistence both within individual hosts and over evolutionary time. This hypothesis could be tested, in part, by an in-depth exploration of variability in the expression and antiviral activity of PHF11 in various tissues and within and between primate and non-primate species.

## Materials and methods

### Cell culture

The adherent human HEK 293T (CVCL_0063), CRFK (CVCL_2426), HT1080 (CVCL_0317), and U3-GFP cell lines were maintained in Dulbecco’s Modified Eagles Medium [(DMEM) Gibco] with 10% fetal calf serum [(FCS) Sigma] and gentamicin (Gibco). The suspension MT4-LTR-GFP cells and U937 (CVCL_0007) lines were maintained in Roswell Park Memorial Institute Medium [(RPMI) Gibco] with 10% FCS and gentamicin. All cells were purchased from ATCC or provided by the NIH AIDS Reagent Program (MT4 parental cells) and were assumed to authenticated by their supplier and were not further characterized. Cells were monitored for retroviral contamination by SYBR-Green based PCR RT assay. Mycoplasma testing was not specifically performed, but these cell lines were routinely used in immunofluorescence assays with Hoechst staining that would have revealed the presence of mycoplasma. HT1080-derived U3-GFP reporter cells were generated by transduction with a pQCXIB-derived MLV-based vector followed by selection in 5μg ml^-1^ blasticidin (Sigma-Aldrich) and single-cell cloning by limiting dilution. Clones were tested for low background and high PFV-sensitive reporter activity by comparing to FAB reporter cells [52]. U3-GFP cells stably expressing PHF11 or myc-tagRFP were generated by transduction with LHCX-derived vectors (Invitrogen) followed by selection in 50μg ml^-1^ hygromycin (ThermoFisher) and single-cell clones derived by limiting dilution. To control the timing of PHF11 expression, derivates of U3-GFP and CRFK cells containing doxycycline-inducible PHF11, were generated by transduction with LKO-derived lentiviral vectors [42] followed by selection in 1μg ml^-1^ puromycin (Sigma-Aldrich). Vector stocks for transduction were generated by co-transfection of 293T cells with a VSV-G expression plasmid, an HIV-1_NL4-3_ Gag-Pol expression plasmid, and an LKO-derived vector, or an MLV Gag-Pol expression plasmid and an LHCX-derived vector using polyethyleneimine (PolySciences). Expression was induced in pLKO transduced cell lines through an overnight treatment (≥ 16 hours) with 500ng/ml doxycycline hyclate (Sigma-Aldrich) prior to challenge with virus or viral vectors. Alternatively, where indicated, doxycycline was added at specific time-points relative to infection and/or the harvesting of cells.

Effects of ectopic PHF11 expression on cellular replication were determined by a cell growth assay. U3-GFP cells stably transduced with doxycycline-inducible myc-HsPHF11 were seeded at a density of 0.5cells/cm^2^ in a 24-well plate in the presence or absence of doxycycline. Every 24 hours until the cells reached confluency (eight days), three wells were trypsinized and live cells were counted using a hemocytometer with trypan blue (Invitrogen) staining to determine cell density. Effects of ectopic PHF11 expression on cell viability were determined by seeding cells at a density of 1.1x10^3^cells/cm^2^ and culturing cells for 11 days in the presence or absence of doxycycline. At days three, six, and nine, cells were passaged at a 1:10 dilution, and the remainder stained for viability using LiveDeadRed (Invitrogen) according to the manufacturer’s protocol and enumerated by FACS analysis using an AttuneNxt cytometer with Autosampler (Life Technologies).

### Viruses

Stocks of replication-competent PFV strains (WT PFV, PFV-rRFP, and PFV-2A-RFP) were generated by transfecting HT1080 cells with 10μg of proviral plasmids using polyethyleneimine and expansion in HT1080 cells for ~48–72 hours. Stocks of SFV_MCY_ (ATCC VR-276) and SFV_AGM_ (ATCC VR-218) were produced by inoculation of HT1080 cells at a high MOI in the presence of a mycoplasma inhibitor (Bio-Rad). Stocks of FFV (VR-1303) were produced by inoculation of CRFK cells at high MOI. FV stocks were harvested when the entire culture was productively infected, as indicated by syncytium formation. Supernatants were harvested, and cell lysates generated by three freeze-thaw cycles, followed by pelleting of cell debris. For spreading infections, inocula were normalized based on levels of reverse transcriptase, determined using a one-step SYBR-Green based PCR RT assay as previously described [53, 54]. Tas-mutant PFV-2A-RFP strains were generated by transfection of 293T cells with 10μg of proviral plasmid. Replication-defective Env-deficient PFV strains were generated by cotransfection of 293T cells with of 10μg of reporter proviral plasmid and 1μg of PFV-Env expression plasmid.

Orthoretrovirus stocks were generated by co-transfection of 293T cells with 10μg of GFP reporter proviral plasmids; HIV-1_NL4-3_ΔEnv-GFP [55], HIV-2_ROD_ΔEnv-GFP, SIV_MAC_ΔEnv-GFP, SIV_AGM_TanΔEnv-GFP [56] and 1μg of VSV-G expression plasmid. For MLV and FIV, three plasmid vector systems [33, 57–59] were also used to generate GFP reporter viruses. In these cases, 5μg of Gag-Pol, 5μg of packagable genome, and 1μg of VSV-G expression plasmids were co-transfected. The CNCR-2A-Tas reporter virus was generated by co-transfection of 5μg of MLV Gag-Pol, 5μg of CNCR-2A-Tas, and 1μg of VSV-G expression plasmids in 293T cells.

The CSGW-based [39] reporter viruses containing reporter cassettes with individual promoter elements driving expression of Tas and RFP were generated by co-transfection of 293T cells with a reporter plasmid, HIV-1_NL4-3_ Gag-Pol expression plasmid, and VSV-G expression plasmid at a 1:1:0.2 ratio. Levels of reverse transcriptase (RT) in viral stocks were quantified using the SYBR-Green based PCR RT assay.

### Plasmid construction

Untagged, and N- or C-terminally Myc-tagged PHF11 expression constructs were generated by PCR amplification and insertion into pLHCX or pLKOΔ using *Sfi*I. PHF11 sequences were amplified from cDNA generated from HT1080 (*Homo sapiens*), NIH3T3 (*Mus musculus domesticus*), or CRFK (*Felis catus*) cells using a SuperScript III reverse transcriptase kit (Life Technologies). pLKOΔ-myc-tagRFP has been previously described [42]. Sequence identity calculations of vertebrate PHF11 genes and amino acid alignments were generated using CLC Main Workbench Software (Qiagen).

The PFV LTR reporter construct (pQXCIB U3-GFP) was generated by overlap-extension PCR of the U3 region of pcPFV [pcHSRV2 [5] kindly provided by Axel Rethwilm] (nucleotide positions 246–794 of GenBank U21247) and GFP (from CSGW). The PCR product was inserted into the retroviral vector pQXCIB [derived from replacement of the neomycin-resistant gene of pQCXIN [60] with the blasticidin-resistance gene using *Bst*XI and *Xho*I] using *Bgl*II. PFV reporter viruses were derived from PFV-rGFP ([61] kindly provided by Maxine Linial) as outlined in S1A Fig. PFV-rRFP was produced by PCR amplification of tagRFP and insertion using *Xma*I. PFV-2A-RFP was produced by PCR amplification of 2A-tagRFP and insertion into PFV-rGFP using *Apa*I and *Xma*I. Fragments of the PFV genome that inactivated Env (ΔEnv) were generated by overlap-extension PCR that introduced silent mutations in the *Pol* reading frame where it overlaps with *Env* to create the following mutations. M1T, M5T, W13STOP, and deleted the remainder *Env* gene 5’ to the IP. The ΔEnv fragment was then inserted into PFV-rRFP and PFV-2A-RFP using *Bst*XI and *Blp*I. RFP was replaced by hygromycin B-resistance genes (PCR amplified from LHCX) using *Xma*I (generating PFVΔEnv-rHygro^R^). To generate a trans-complementing Env expression plasmid, PFV *Env* was PCR amplified from PFV-rGFP [61] (nucleotide positions 6498–9464 of GenBank KX087159) and cloned into pCAGGS using *Xho*I and *Not*I.

A derivative of PFV-2A-RFP that expressed Tas containing the W279R mutation was generated by PCR mutagenesis, Derivatives of PFV-2A-RFP expressing Tas_W279R_-transactivator fusions [VP16 amino acids 410–490 and HIV-1 Tat amino acids 1–48 [37]] were constructed by generating overlap-extension Tas_W279R_-VP16 and Tas_W279R_-Tat PCR products that were then inserted into PFV-2A-RFP using *Blp*I and *Apa*I.

CNCR-2A-Tas was derived from the MLV reporter construct pCNCG by PCR amplification of tagRFP, with the addition of a 3’ 2A cleavage site and *Not*I site and insertion into pCNCG using *Age*I and *Hpa*I, followed by insertion of PFV Tas using *Not*I and *Hpa*I.

CSGW-based reporter constructs were generated by replacing the SFFV promoter with the PFV U3 (from pQXCIB U3-GFP) or IP promoter (PCR amplified from pcPFV, nucleotide positions 8970–9249 of GenBank U21247) and insertion into pCSGW using *EcoR*I and *Tth111*I. Thereafter RFP, Tas-RSV-RFP, or Tas-2A-RFP reporters (PCR amplified from PFV reporter constructs described above) were inserted using *Tth111*I and *Xho*I.

### Infection assays

Single-cycle infectivity was measured using U3-GFP cells seeded in 96-well plates at 5 x 10^3^ cells per well and inoculated with serial-dilutions of virus. Where indicated, cells were pretreated with doxycycline for ≥ 16 hours prior to infection. Two days post-infection, cells were trypsinized and fixed in 2% paraformaldehyde. In some experiments, cells were treated with 10μM raltegravir (Sigma-Aldrich) at the time of infection, or at specific time-points following infection. Infected cells (%GFP, %RFP, and %GFP of RFP-positive cells) were enumerated by FACS analysis using a CyFlow cytometer (Partec) coupled to a Hypercyte Autosampler (Intellicyt) or an AttuneNxt cytometer with Autosampler (Life Technologies). Spreading replication assays were conducted by seeding cells in 24 or 48 well plates at 2.5 x 10^4^ or 1.25 x 10^4^ cells per well, respectively. At 16–24 hours later, cells were infected with PFV at a MOI of 0.001 to 0.05, or equivalent amounts of SFV_MCY_, SFV_AGM_, or FFV_FCA_ as determined by SYBR-Green RT assay. The next day, cells were washed three times and supernatant samples were harvested every 1 or 2 days. Cells were split 1:5 as needed, and when replication was measured by GFP expression from the U3-reporter, a portion of the remaining cells were fixed in 2% paraformaldehyde and enumerated by flow cytometry. PFV integration was measured by transduction of U3-GFP cells seeded in 12 well plates at 5 x 10^4^ cells per well with or without ≥ 16 hours of pre-treatment with doxycycline, or in the presence of raltegravir (added at the time of infection). Cells were trypsinized and re-plated in 10cm dishes two days after infection and selected in 50μg ml^-1^ hygromycin for 10–14 days. Hygromycin-resistant colonies were stained with crystal violet (1% crystal violet, 20% ethanol in dH_2_O) and counted. For spreading infection, or hygromycin-resistant colony formation assays, cells were maintained in media with or without doxycycline for the duration of the experiment.

Effects of PHF11 on expression from CSGW-based reporter constructs were measured by transducing U3-GFP cells containing doxycycline-inducible myc-HsPHF11 constructs with or without ≥ 16 hours of pretreatment with doxycycline. Viral inocula were normalized for RT activity. Two days following transduction, cells were trypsinized and fixed in 2% paraformaldehyde and the percentage of RFP positive and GFP positive cells were enumerated by FACS analysis.

### Western blotting

Cell suspensions were lysed in NuPage LDS sample buffer (Novex), followed by sonication, separated by electrophoresis on NuPage 4–12% Bis-Tris gels (Novex) and blotted onto polyvinylidene fluoride (PDVF, BioRad Laboratories). Membranes were incubated with antibodies against the Myc tag (Millipore 05–724), LaminB1 (Abcam ab133741), Tubulin (Sigma-Aldrich T6074), or PHF11 (Proteintech 10898-1-AP) followed by incubation with goat anti-rabbit-HRP or goat anti-mouse-HRP secondary antibodies (Jackson ImmunoResearch). SeeBlue Pre-stained Protein Standard (Thermo Fisher) was used for protein size determination. Blots were developed with SuperSignal West Femto Maximum Sensitivity Substrate (Thermo Scientific) and imaged/quantified on a C-Digit scanner (LI-COR Biosciences). For detection of endogenous PHF11 expression, MT4-LTR-GFP cells and U937 cells were pre-treated with 1000U/ml universal type-I IFN (PBL assay science) for 24 hours prior to lysis. The predicted molecular weights of the PHF11 proteins are as follows: human, 37.6 kDa; macaque 37.6 kDa; feline, 39.7 kDa; mouse A, 32.3 kDa; mouse B, 30.0 kDa; mouse C, 38.1 kDa; and mouse D, 36.0 kDa. The location of the Myc tag (1.7kDa) appears to alter the apparent molecular weight of PHF11 but not its antiviral activity [MacPHF11-myc runs at a slightly higher molecular weight than HsPHF11-myc (Fig 1A), while the opposite is true for N-terminally tagged human and macaque PHF11 (Fig 3), but all four proteins possess similar antiviral activity].

### Statistical analysis

Statistical tests were implemented using GraphPad Prism software (two-tailed parametric t-test). Where appropriate (e.g. comparison of doxycycline treated and untreated samples infected with the same virus dilution) a paired t-test was utilized. For comparison of PHF11 activity on CSGW-based reporter constructs, percentage of cells expressing GFP or RFP in the absence or presence of doxycycline (S6 Fig) from two experiments utilizing the same viral inocula (as determined by RT levels) were converted to a fold-change (Fig 5B–5D) before statistical analysis.

## Supporting information

S1 Fig PFV reporter cells. (A) Schematics of PFV reporter constructs utilized in U3-GFP cells. (B) Sample FACS plots illustrating the GFP and RFP expression in U3-GFP cells that were uninfected or infected with WT PFV.
(TIF)

S2 Fig PHF11 expression does not affect cell growth or viability. U3-GFP cells stably transduced with doxycycline-inducible myc-HsPHF11 were cultured in the presence or absence of doxycycline for the indicated length of time. (A) Cells were plated at a density of 0.5cells/cm^2^ and counted every 24 hours until fully confluent. (B) Cells were plated at a density of 1.1x10^3^cells/cm^2^, passaged at a 1:10 dilution at the indicated time-points, and the remainder stained for viability with LiveDeadRed followed by flow cytometry analysis. n = 3, technical replicates.
(TIF)

S3 Fig A 48-hour infection of PFV in HT1080 U3-GFP cells is a single cycle of infection. U3-GFP cells were infected with PFV in the presence or absence of 10μM raltegravir at the time of infection, or with raltegravir added 24 hours post-infection. Viral titer was determined by percentage of GFP positive cells are represented as mean + sem of infectious units per ml, n≥3 technical replicates.
(TIF)

S4 Fig Expression of endogenous PHF11. Western blot analysis of PHF11 expression in IFN-treated U3-GFP MT4-LTR-GFP (MT4-GFP), U937 cells, or doxycycline inducible U3-GFP cells and lamin B1 loading control.
(TIF)

S5 Fig Expression of mammalian PHF11 genes and activity in single-cycle infection assays. (A) Single-cycle PFV infection U3-GFP cells expressing doxycycline-inducible myc-PHF11 in the presence (white bars) or absence (black bars) of 16-hour pretreatment with doxycycline (Dox). (B) Single-cycle PFV infection of U3-GFP cells expressing doxycycline-inducible myc-PHF11 in the presence (white bars) or absence (black bars) of pretreatment with doxycycline (Dox). (C) Top: diagram of human, feline, and chimeric PHF11 proteins with amino acid lengths and domain locations indicated. NTD = N-terminal domain; PHD = extended PHD finger; CTD = C-terminal domain. (D) Western blot analysis of indicated doxycycline inducible myc-PHF11 proteins and LaminB1 loading control in U3-GFP cells. (E) Single-cycle PFV infection of U3-GFP cells expressing doxycycline-inducible myc-PHF11 in the presence (white bars) or absence (black bars) of pretreatment with doxycycline (Dox). Titers were determined by percentage of GFP positive cells and are represented as mean + sem of infectious units per ml, n≥3 technical replicates. Representative of at least three independent experiments.
(TIF)

S6 Fig PHF11 inhibits expression from the FV internal promoter. Transduction of U3-GFP expressing doxycycline-inducible myc-HsPHF11 in the presence (open circles) or absence (filled circles) of doxycycline (Dox) pretreatment. Left panels: Promoters directing Tas expression and RFP expression and diagrams of promoter, viral, and reporter elements color-coded as shown below and in Fig 7. Middle panels: Percentage of RFP positive cells at indicated inocula defined by units of reverse transcriptase (RT). Right panels: Percentage of GFP positive cells. Blue lines, SFFV promoter; green lines, PFV-U3 promoter; purple lines, PFV-IP. Representative of four independent experiments.
(TIF)
