# Supplementary figures and images for: Inhibition of spumavirus gene expression by PHF11
Source: PLoS Pathog. 2020 Jul 17;16(7):e1008644. doi: 10.1371/journal.ppat.1008644 (PMC7390438; doi:10.1371/journal.ppat.1008644)

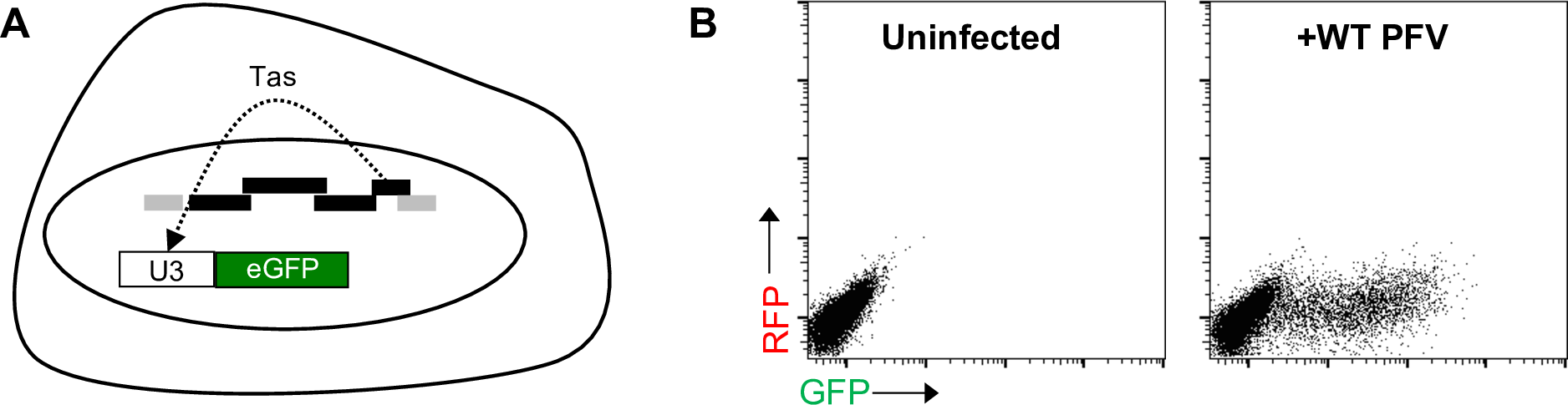

Supplement: S1 Fig — (A) Schematics of PFV reporter constructs utilized in U3-GFP cells. (B) Sample FACS plots illustrating the GFP and RFP expression in U3-GFP cells that were uninfected or infected with WT PFV. (TIF) [file ppat.1008644.s001.tif]

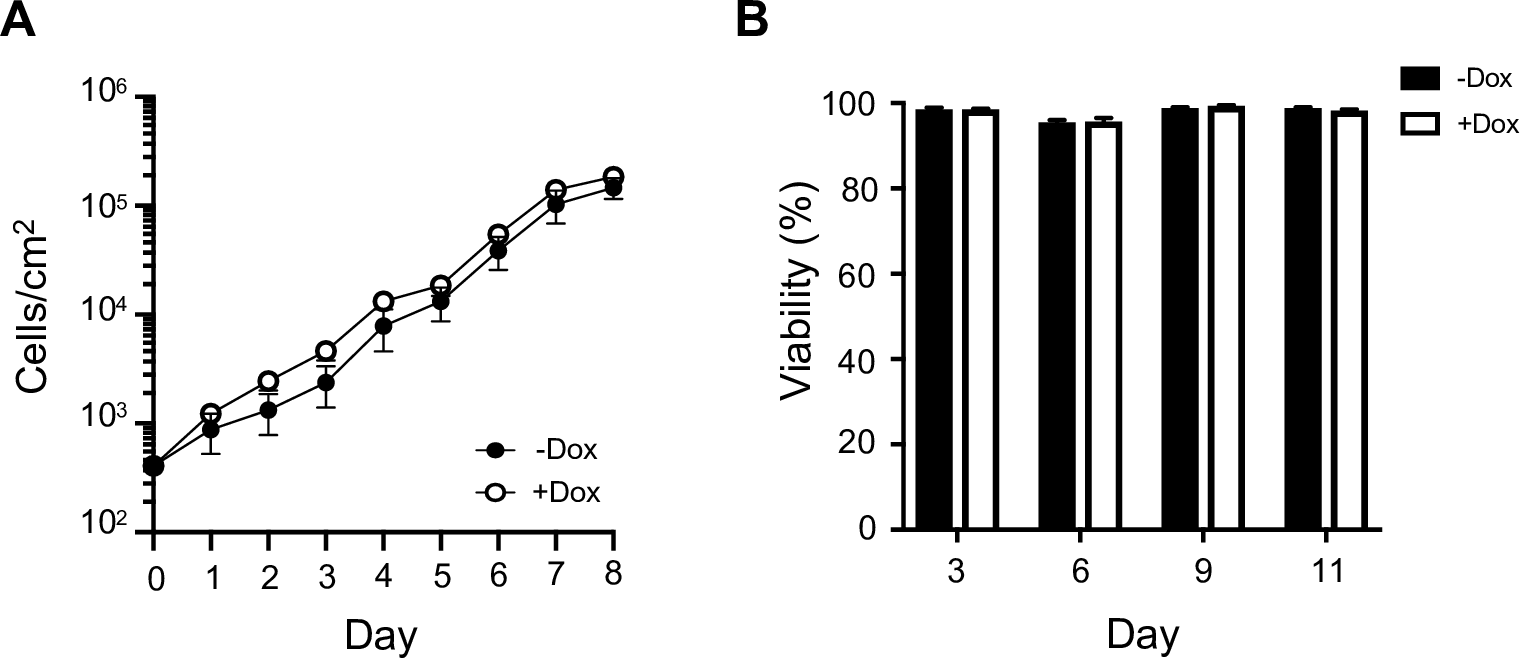

Supplement: S2 Fig — U3-GFP cells stably transduced with doxycycline-inducible myc-HsPHF11 were cultured in the presence or absence of doxycycline for the indicated length of time. (A) Cells were plated at a density of 0.5cells/cm2 and counted every 24 hours until fully confluent. (B) Cells were plated at a density of 1.1x10^3^cells/cm2, passaged at a 1:10 dilution at the indicated time-points, and the remainder stained for viability with LiveDeadRed followed by flow cytometry analysis. n = 3, technical replicates. (TIF) [file ppat.1008644.s002.tif]

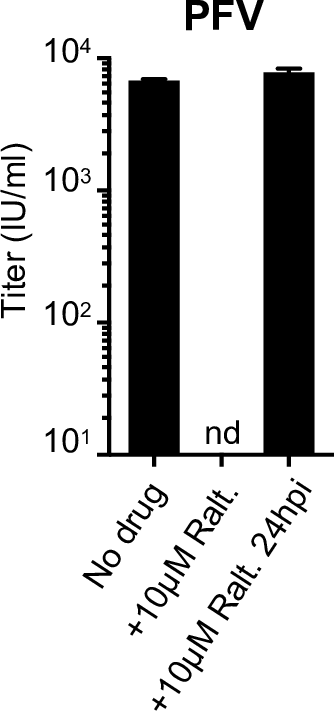

Supplement: S3 Fig — U3-GFP cells were infected with PFV in the presence or absence of 10μM raltegravir at the time of infection, or with raltegravir added 24 hours post-infection. Viral titer was determined by percentage of GFP positive cells are represented as mean + sem of infectious units per ml, n≥3 technical replicates. (TIF) [file ppat.1008644.s003.tif]

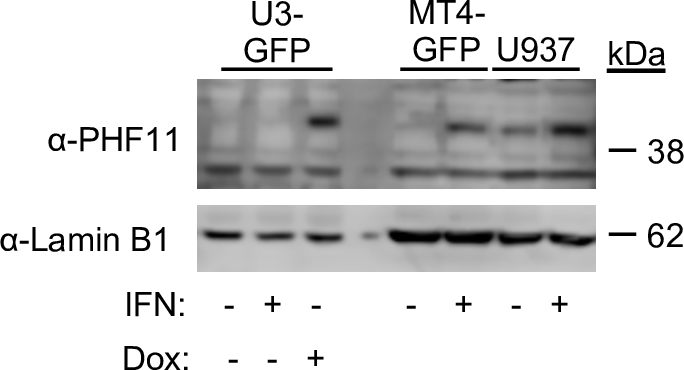

Supplement: S4 Fig — Western blot analysis of PHF11 expression in IFN-treated U3-GFP MT4-LTR-GFP (MT4-GFP), U937 cells, or doxycycline inducible U3-GFP cells and lamin B1 loading control. (TIF) [file ppat.1008644.s004.tif]

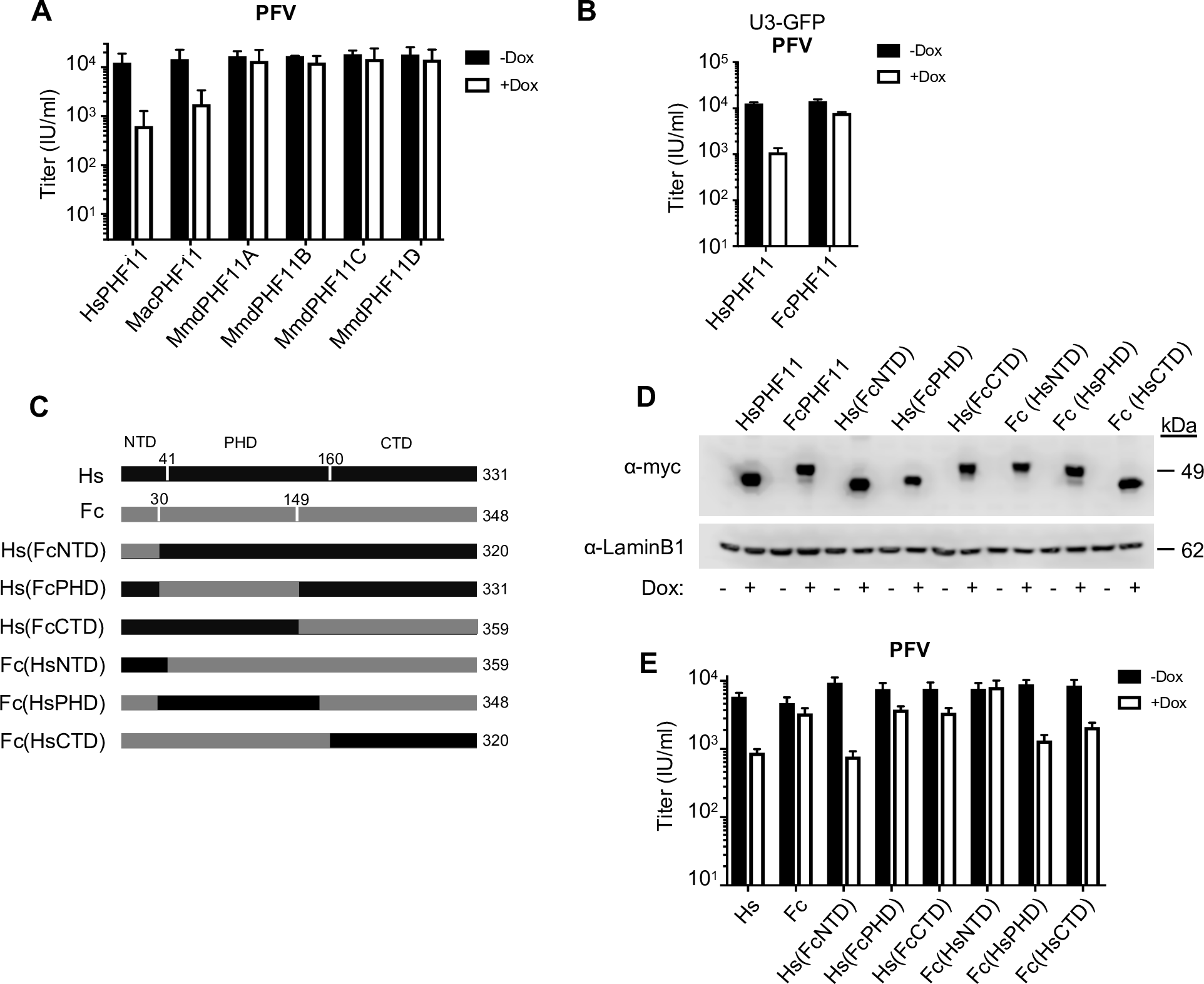

Supplement: S5 Fig — (A) Single-cycle PFV infection U3-GFP cells expressing doxycycline-inducible myc-PHF11 in the presence (white bars) or absence (black bars) of 16-hour pretreatment with doxycycline (Dox). (B) Single-cycle PFV infection of U3-GFP cells expressing doxycycline-inducible myc-PHF11 in the presence (white bars) or absence (black bars) of pretreatment with doxycycline (Dox). (C) Top: diagram of human, feline, and chimeric PHF11 proteins with amino acid lengths and domain locations indicated. NTD = N-terminal domain; PHD = extended PHD finger; CTD = C-terminal domain. (D) Western blot analysis of indicated doxycycline inducible myc-PHF11 proteins and LaminB1 loading control in U3-GFP cells. (E) Single-cycle PFV infection of U3-GFP cells expressing doxycycline-inducible myc-PHF11 in the presence (white bars) or absence (black bars) of pretreatment with doxycycline (Dox). Titers were determined by percentage of GFP positive cells and are represented as mean + sem of infectious units per ml, n≥3 technical replicates. Representative of at least three independent experiments. (TIF) [file ppat.1008644.s005.tif]

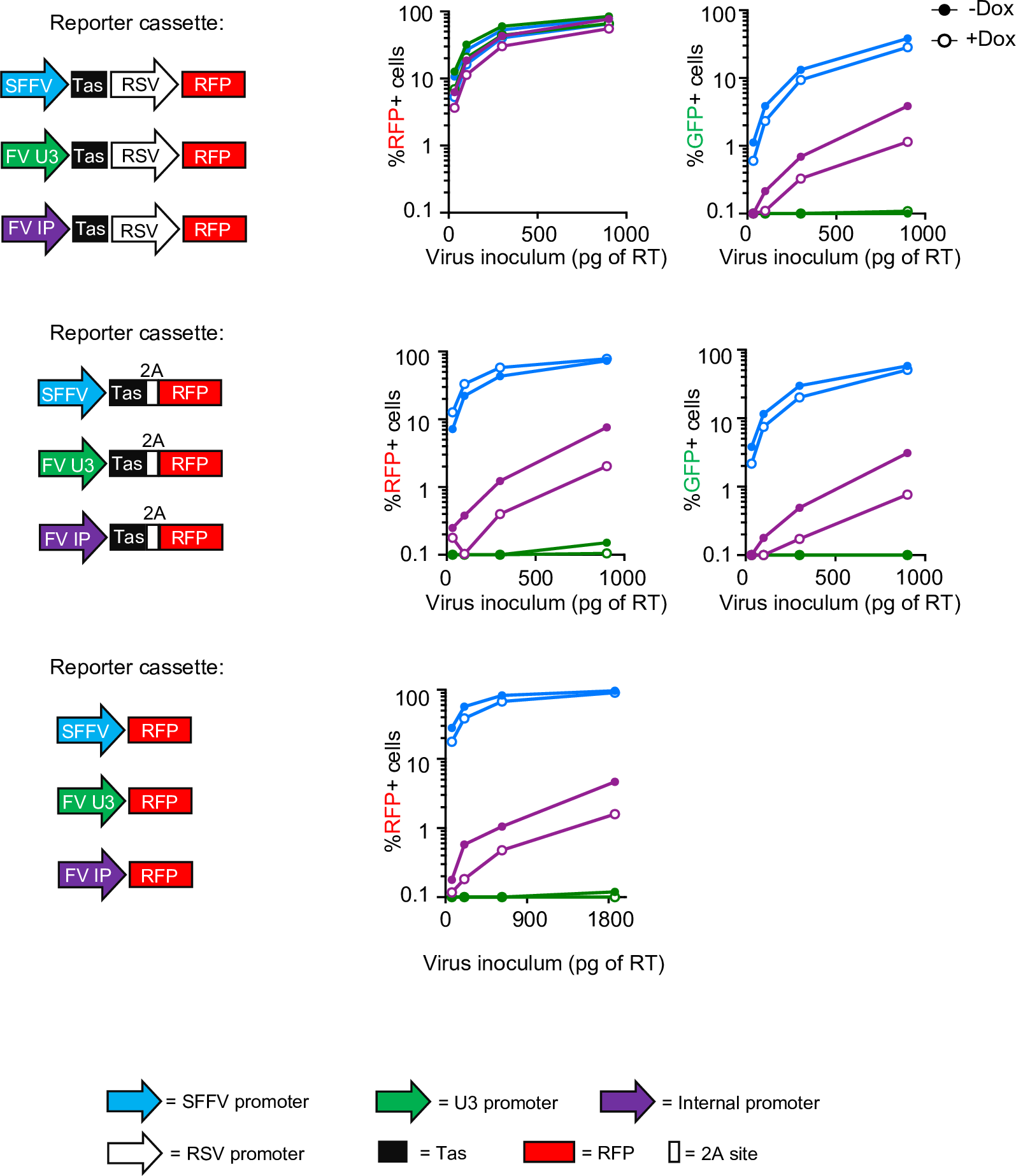

Supplement: S6 Fig — Transduction of U3-GFP expressing doxycycline-inducible myc-HsPHF11 in the presence (open circles) or absence (filled circles) of doxycycline (Dox) pretreatment. Left panels: Promoters directing Tas expression and RFP expression and diagrams of promoter, viral, and reporter elements color-coded as shown below and in Fig 7. Middle panels: Percentage of RFP positive cells at indicated inocula defined by units of reverse transcriptase (RT). Right panels: Percentage of GFP positive cells. Blue lines, SFFV promoter; green lines, PFV-U3 promoter; purple lines, PFV-IP. Representative of four independent experiments. (TIF) [file ppat.1008644.s006.tif]
